# Genome-wide transcriptome profiling of nitrogen fixation in *Paenibacillus* sp. WLY78

**DOI:** 10.1186/s12866-016-0642-6

**Published:** 2016-03-01

**Authors:** Hao-wen Shi, Li-ying Wang, Xin-xin Li, Xiao-meng Liu, Tian-yi Hao, Xiao-juan He, San-feng Chen

**Affiliations:** State Key Laboratory for Agrobiotechnology and Key laboratory of Soil Microbiology of Agriculture Ministry, China Agricultural University, Yuanmingyuan west road No.2, Haidian District Beijing, 100193 P. R. China

**Keywords:** *Paenibacillus* sp. WLY78, *nif* gene, Transcription, Up-regulation, Nitrogen metabolism

## Abstract

**Background:**

Diazotrophic (nitrogen-fixing) Gram-positive and endospore-formed *Paenibacillus* spp. have potential uses as a bacterial fertilizer in agriculture. The transcriptional analysis of nitrogen fixation in *Paenibacillus* is lacking, although regulation mechanisms of nitrogen fixation have been well studied in Gram-negative diazotrophs.

**Results:**

Here we report a global transcriptional profiling analysis of nitrogen fixation in *Paenibacillus* sp. WLY78 cultured under N_2_-fixing condition (without O_2_ and NH_4_^+^) and non-N_2_-fixing condition (air and 100 mM NH_4_^+^). The *nif* (nitrogen fixation) gene operon composed of 9 genes (*nifBHDKENXhesAnifV*) in this bacterium was significantly up-regulated in N_2_-fixing condition compared to non-N_2_-fixing condition, indicating that *nif* gene transcription is strictly controlled by NH_4_^+^ and O_2_. qRT-PCR confirmed that these *nif* genes were differently expressed. Non-*nif* genes specifically required in nitrogen fixation, such as *mod*, *feoAB* and *cys* encoding transporters of Mo, Fe and S atoms, were coordinately transcribed with *nif* genes in N_2_-fixing condition. The transcript abundance of *suf* operon specific for synthesis of Fe-S cluster was up-regulated in N_2_-fixing condition, suggesting that Sul system, which takes place of *nifS* and *nifU*, plays important role in the synthesis of nitrogenase. We discover potential specific electron transporters which might provide electron from Fe protein to MoFe protein of nitrogenase. The *glnR* whose predicted protein might mediate *nif* transcription regulation by NH_4_^+^ is significantly up-regulated in N_2_-fixing condition. The transcription levels of nitrogen metabolism and anaerobic respiration were also analyzed.

**Conclusions:**

The *nif* gene operon (*nifBHDKENXhesAnifV*) in *Paenibacillus* sp. WLY78 is significantly up-regulated in N_2_-fixing condition compared to non-N_2_-fixing condition. Non-*nif* genes specifically required in nitrogen fixation were also significantly up-regulated in N_2_-fixing condition. Fur and Fnr which are involved in anaerobic regulation and GlnR which might mediate *nif* gene transcription regulation by NH_4_^+^ were significantly up-regulated in N_2_-fixing condition. This study provides valuable insights into nitrogen fixation process and regulation in Gram-positive firmicutes.

**Electronic supplementary material:**

The online version of this article (doi:10.1186/s12866-016-0642-6) contains supplementary material, which is available to authorized users.

## Background

Biological nitrogen fixation, the conversion of atmospheric N_2_ to NH_3_, plays an important role in the global nitrogen cycle and in world agriculture [1]. The ability to fix nitrogen is widely, but sporadically distributed among Archaea and Bacteria which includes these families: Proteobacteria, Firmicutes, Cyanobacteria, Actinobacteria and Chlorobi [2–5]. The *nif* gene number and organization vary greatly among diazotrophs [6–13]. For example, twenty *nif* genes, *nifJHDKTYENXUSVWZMFLABQ*, organized in several transcriptional units, are clustered in a single 23-kb region in the chromosome of *Klebsiella oxytoca* [8]*.* Genetic and biochemical studies on the two model diazotrophs (*K. oxytoca* and *Azotobacter vinelandii*) revealed that 16 *nif* gene (*nifH, D, K, Y, T*, *E, N, X, U, S, V, Z, W, M, B, Q*) products are probably essential for efficient biosynthesis of nitrogenase [3, 14]. In addition to those genes specifically required for the biosynthesis and activity of nitrogenase, the non-*nif* genes encoding transporters for Mo, Fe and S play important roles in nitrogen fixation. Almost all of the *nif* genes from Gram-negative diazotrophs possess a σ^54^-dependent promoter which requires a form of RNA polymerase holoenzyme containing a unique sigma factor, σ^N^ (σ^54^) encoded by the *rpoN* gene. Transcription of *nif* genes in these diazotrophs is stringently regulated in response to environmental oxygen and ammonium. In *K. oxytoc*a, *nif* genes are subject to two levels of regulation, one global and the other *nif* specific. The *nif*-specific regulation is mediated by the NifA (*nifA* gene product) which is a transcriptional activator required for the expression of all *K. oxytoc*a *nif* operons, except its own [15]. The global level of *nif* regulation in *K. oxytoc*a is mediated by the global nitrogen regulator NtrC.

In contrast to these Gram-negative diazotrophs, *Paenibacillus* sp. WLY78, a Gram-positive bacterium, possesses a minimal and compact *nif* gene cluster consisting of 9 genes (*nifBnifHnifDnifKnifEnifNnifXhesAnifV*) [16]. The 9 *nif* genes are organized as an operon and possess a σ^70^-dependent promoter located in front of *nifB* gene. The genome of *Paenibacillus* sp. WLY78 does not have *nifA* [16]. The nitrogease activity of *Paenibacillus* sp. WLY78 was inhibited by high concentration of NH_4_^+^ and O_2_ [16]. These data suggest that regulation mechanisms of nitrogen fixation differ greatly between Gram-positive *Paenibacillus* and Gram-negative *K. oxytoca* and *A. vinelandii* [17, 18].

Here we performed genome-wide transcription profiling analysis of *Paenibacillus* sp. WLY78 cultured under N_2_-fixing (without O_2_ and NH_4_^+^) and non-N_2_-fixing (air and 100 mM NH_4_^+^) conditions. Our results revealed that the *nif* genes and non-*nif* genes specifically required for nitrogen fixation in *Paenibacillus* were coordinately expressed in N_2_-fixing condition compared to non-N_2_-fixing condition. The transcription levels of nitrogen metabolism and anaerobic respiration were also analyzed. Our study provides valuable insights into nitrogen fixation process and regulation of Gram-positive *Paenibacillus*.

## Results

### Genome-wide transcription analysis of *Paenibacillus* sp. WLY78

**Fig. 1:**
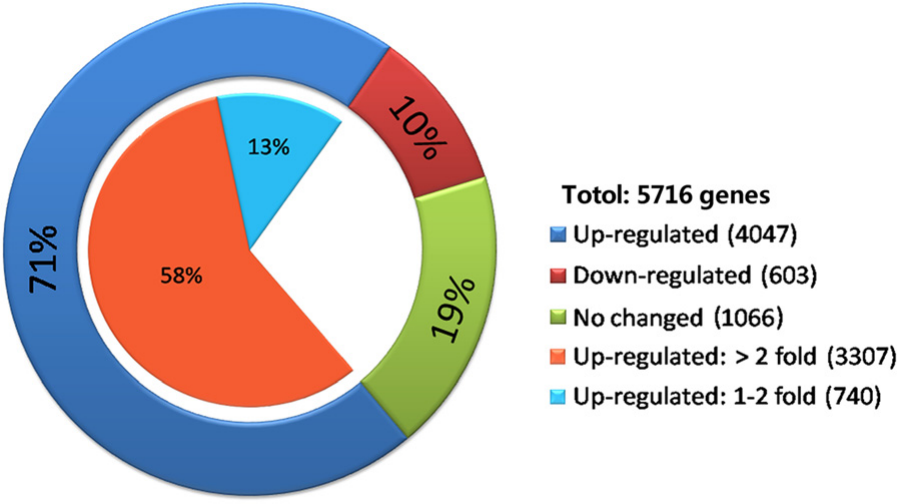
Changes in transcript levels of the total genes within the *Paenibacillus* sp. WLY78 genome

A genome-wide transcription analysis of the nitrogen-fixer *Paenibacillus* sp. WLY78 cultured under N_2_-fixing and non-N_2_-fixing conditions was performed. Among 5716 genes of *Paenibacillus* sp.WLY78, transcript abundances were increased for 4047 genes (71 %), decreased for 603 genes (10 %) and not changed for 1066 genes (19 %) under N_2_-fixing condition compared to non-nitrogen-fixing condition control (Fig. 1 and Additional file 1: Table S1). Based on log_2_ fold changes (*p* < 0.05), transcript levels for nearly 60 % (3307 among 5716 genes) of the *Paenibacillus* sp. WLY78 genes changed more than 2-fold under N_2_-fixing condition relative to those under the non-N_2_-fixing condition control.

### Transcriptional analysis of the nitrogen fixation genes

**Fig. 2:**
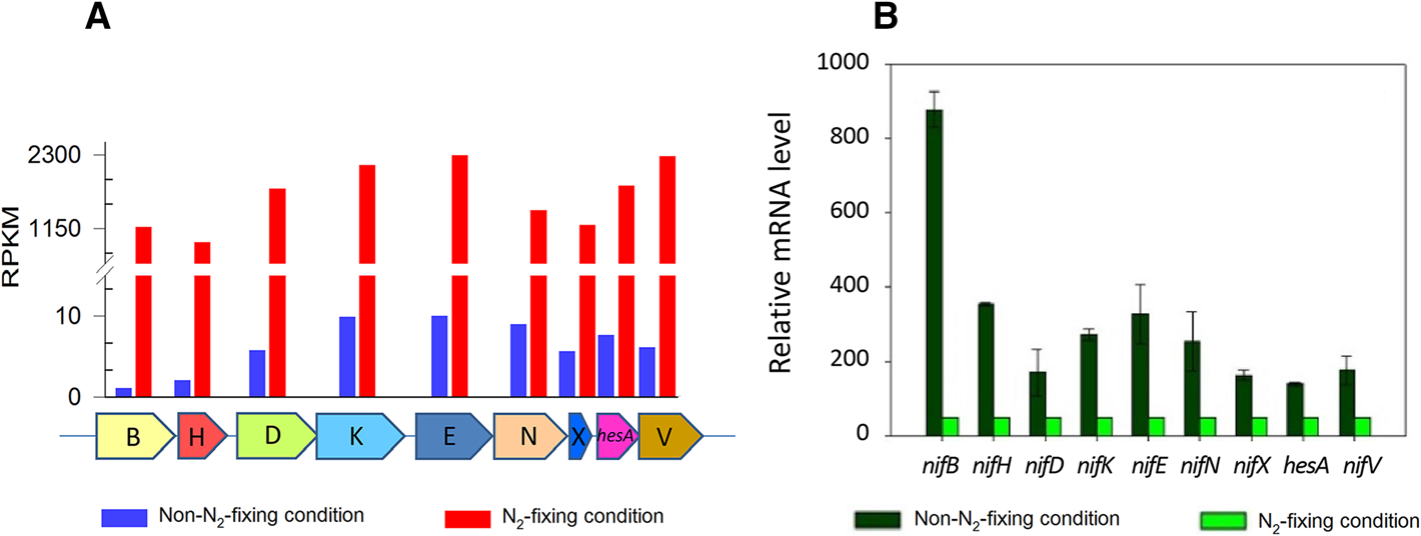
Differential expression of the *Paenibacillus nif* genes in N_2_-fixing and non-N_2_-fixing conditions. **a** Transcriptional analysis of the *nif* genes. **b** Quantitative real-time RT-PCR analysis of *nif* gene expression

The expression levels of the 9 genes *nifBHDKENXhesAnifV* in *Paenibacillus* sp. WLY78 fall into the range of the first two top expression levels under N_2_-fixing condition, while they fall into the range of the low expression levels under non-N_2_-fixing condition (Table 1). These 9 genes within the *nif* operon exhibited a significant transcript increase ranging from 150- to 1039-fold in N_2_-fixing condition compared to non-N_2_-fixing condition, suggesting that *nif* gene expression in *Paenibacillus* was strongly regulated by ammonium and oxygen (Fig. 2a and Additional file 1: Table S1). qRT-PCR also confirmed that the 9 genes within the *nif* operon were significantly differently expressed in N_2_-fixing condition compared to non-N_2_-fixing condition (Fig. 2b). Furthermore, we found that the transcript abundances varied greatly among these 9 *nif* genes although all of them were cotranscribed from a common promoter. The different abundances of these cotranscribed genes suggested that these transcripts have different processing and stabilities, similar results also were found in *A. vinelandii* [19]. The transcriptional profile of *Paenibacillus* sp. WLY78 is a little different from that of *A. vinelandii* where the transcript level of *nifH* were much higher than those of *nifD*, *nifK* and the other remaining genes [19].

**Table 1:** Relative expression level (Reads Per Kilobase per Million, RPKM) in *Paenibacillus* sp. WLY78

RPKM	Gene numbers/*Paenibacillus* sp. WLY78	
	Non-N_2_-fixation (100 mM NH_4_^+^ and Air)	N_2_-fixation (without NH_4_^+^ and O_2_)
0	100	15
0--0.25(>0)	54	25
0.25--0.5	271	37
0.5--1	649	132
1.0--5.0	2294	868
5.0--10	705^a^	592
10--50	1082	1787
50--100	215	809
100--500	284	1108
500--1000	48	184^a^
1000--∞	27	172^a^

^a^The gene number of this relative expression level includes the *nif* genes

### Transcriptional analysis of molybdate transporters

Molybdenum is essential in bacteria for the activity of a limited number of microbial enzymes [20], including nitrogenase [21] and nitrate reductase [22]. As molybdate is present in the environment in only trace amounts, bacteria employ an energy-dependent high-affinity molybdate transporter to accumulate it. Molybdate is transported mainly by the high-affinity ModABC system. It was reported that the CysPTWA (SulT) sulfate-thiosulfate permease, which transports sulfate, also can transport molybdenum with lower affinity that requires high molybdate concentrations [23].

**Fig. 3:**
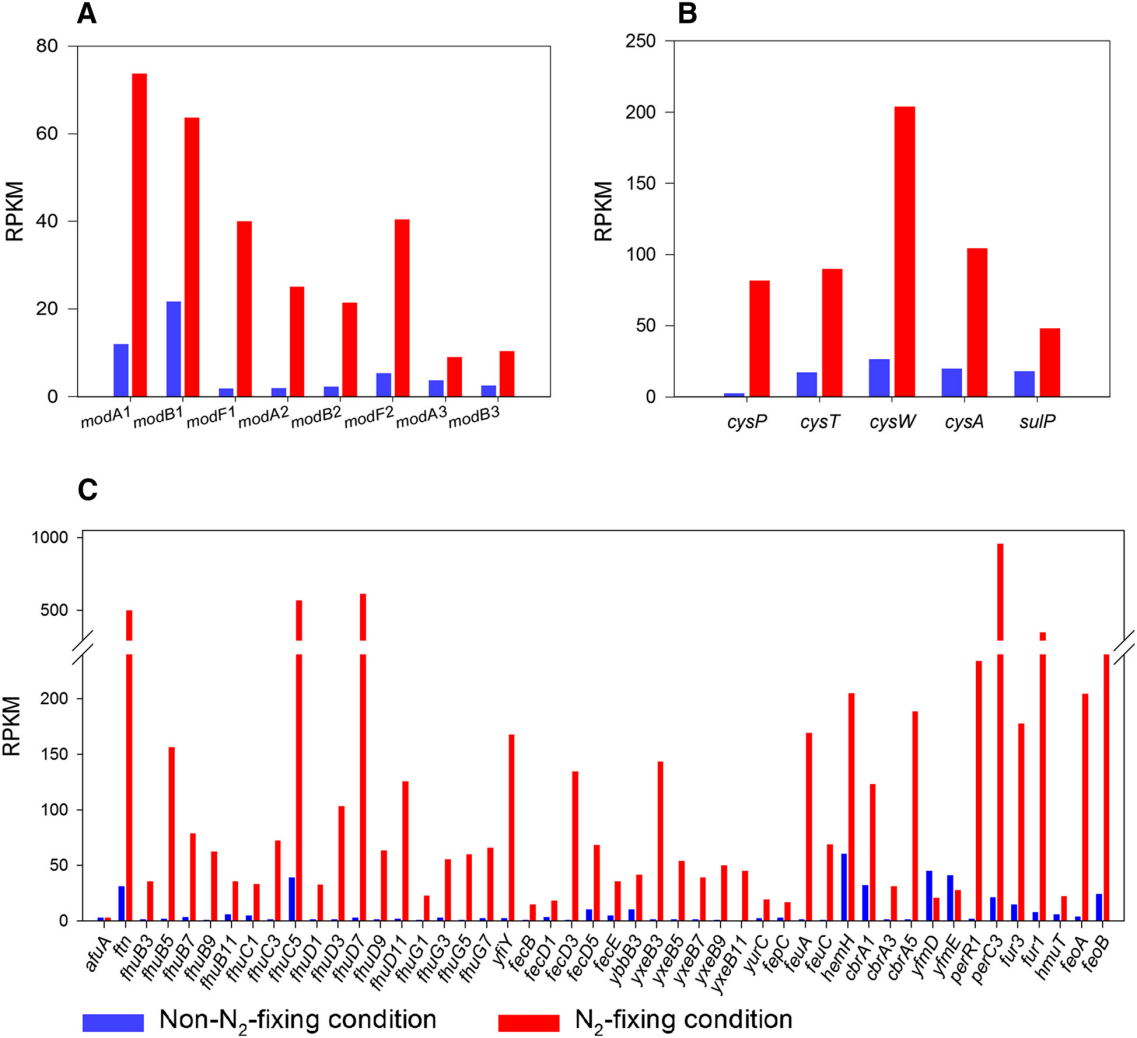
Differential expression of the genes relating to the transport, storage and regulation of molybdenum, sulfate and iron in N_2_-fixing and non-N_2_-fixing conditions. **a** The *mod* genes encoding molybdenum transporters. **b** The genes encoding sulfate transporters. **c** The genes encoding iron transport, storage and regulation

Here we find that *Paenibacillus* sp. WLY78 has 10 *mod* genes including *modA1B1CF1, modA2B2F2, modA3B3* and COG1910 (encoding periplasmic molybdate-binding protein). Except for *modC* being down-regulated, the expression levels of other 9 *mod* genes were up-regulated from about 2-fold to 21-fold in N_2_-fixing condition compared to non- N_2_-fixing condition (Fig. 3a and Additional file 1: Table S2). The data suggest that transcriptions of molybdate transporters were coordinately induced with *nif* genes in *Paenibacillus* sp.WLY78 under N_*2*_-fixing condition.

### Transcriptional analyis of sulfate transporters

Sulfur is an essential element for microorganisms, especially for diazotrophs whose nitrogenase contains iron-sulfur clusters [3–5, 23]. Sulfur can be obtained from varied compounds, sulfate (SO_4_^2−^) and thiosulfate (S_2_O_3_^2−^) being the preferred sulfur sources for the majority of organisms. Sulfate and thiosulfate are taken up by membrane transporters called sulfate permeases. Bacterial sulfate permeases belong to the SulT (Sbp/CysPTWA), SulP, CysP/(PiT) and CysZ families [23]. It is reported that sulfate is structurally related to the oxyanions molybdate, and it can also be transported by the ModABC molybdate transport system [22].

Sulfate permeases in *Paenibacillus* sp. WLY78 include the SulT (SbpCysTWA), SulP and CysP/(PiT). The SulT (sulfate-thiosulfate) permease of *Paenibacillus* sp. WLY78 was constituted by *sbp*, *cysT*, *cysW* and *cysA* gene products. The *sbp* and *cysTW* form an operon, while *cysA* is located in another chromosomal region. The *Paenibacillus* CysP sulfate permease, which is similar to CysP of *B. subtilis*, belongs to the PiT family of phosphate transporters, and may also transport sulfate. As shown in Fig. 3b and Additional file 1: Table S3, *sbpcysTWA* (encoding the SulT), s*ulP* (encoding SulP), and *cysP* (encoding CysP/(PiT)) in *Paenibacillus* sp. WLY78 were up-regulated from 2.5-fold to 32.9-fold. We also find that Sul1, which is a putative sulfate permease, was up-regulated in N_2_-fixing condition. In addition, the *cysK1, cysK3*, *cysK5* and *cysK7* encoding cysteine synthase were up-regulated, and *cysH, cysS, cysC, cysE, cysG*, *cysI* and *cysJ* genes involved in sulfur metabolism were also up-regulated in N_2_-fixing condition compared to non-N_2_-xondition.

### Transcriptional analyis of Fe transporter

Iron (Fe) is an essential element for almost all organisms and is required in cofactors for many enzymes, including nitrogenase. At neutral pH, iron is often biologically unavailable because of the poor solubility of ferric iron [24]. Many bacteria excrete ferric chelators, known as siderophores, to take up ferric iron (Fe^3+^). Usually, bacteria take up ferric complexes (e.g. ferri-siderophores, haem and haem–protein complexes, ferric–transferrin/lactoferrin complexes, and ferric–citrate) [25]. At acidic pH or under anaerobic condition, Fe is the soluble Fe^2+^ form (ferrous iron). The major route for bacterial-ferrous-iron uptake would appear to be, in many cases, via Feo (Ferrous iron transport) [26]. Enterobacterial Feo systems are composed of three proteins: FeoA, FeoB and FeoC. FeoB is responsible for ferrous iron transport, but the functions of FeoA and FeoC remain unclear. The *feoABC* genes constitute an operon. However, the *feoA* and *feoC* genes are not always present alongside *feoB* in some bacteria [26, 27].

Our study reveals that there are 48 Fe transporter genes in the genome of *Paenibacillus* sp. WLY78, indicating this bacterium is very rich in the Fe transporter (Fig. 3c and Additional file 1: Table S4). Except for three genes *yfmD* (Fe^3+^siderophore ABC-transporter permease), *yfmE* (Fe^3+^dicitrate ABC-transporter permease) and *ftpA (*ABC-type Fe^3+^ transport system) being weakly down-regulated, the other 45 genes were up-regulated from 1- to 205-fold in N_2_-fixing condition compared to non-N_2_-fixing condition. Notably, of the 48 Fe transporter genes, 41 genes belong to Fe^3+^ transport systems including Fe^3+^ siderophores transport systems and Fe^3+^ hydroxamate transport systems, and 6 genes were involved in Fe^2+^ uptake and regulation and 1 gene (*ftn*) encodes iron storage. The highest expressed Fe transporter in N_2_-fixing condition was *fhuD7* encoding Fe^3+^ transporter. Feo system of *Paenibacillus* sp. WLY78 is composed of *FeoA* and *FeoB* and responsible for uptake of Fe^2+^. *feoAB* were up-regulated 54- and 12-fold, respectively, in N_2_-fixing condition. *fit* gene encoding iron storage protein was up-regulated 16-fold. Notably, *Paenibacillus* contains 2 *fur* genes*,* which were up-regulated 43- and 11-fold, respectively*.* The *fur* gene codes for the transcriptional activator Fur (Ferric uptake regulator), which controls its own synthesis as well as the transcription of genes involved in the iron homeostasis [28, 29]. It also participates in the regulation of other cellular functions such as oxidative stress, glycolysis, TCA cycle, respiration, 2, 3-dihydroxybenzoate biosynthesis [28, 29]. In addition to *fur* genes, *Paenibacillus* has 2 genes *perC3* and *perR1* encoding Fe^2+^/Zn^2+^ uptake regulation proteins and they were significantly up-regulated 44- and 108-fold, respectively, in N_2_-fixing condition compared to non-N_2_-fixing condition. These data indicate that both Fe^2+^ and Fe^3+^ uptake play important role in nitrogen fixation of *Paenibacillus* sp. WLY78. Especially, Fe^3+^ transporters may play a major role in nitrogen fixation of *Paenibacillus* sp. WLY78, in accordance with that bacterium is grown in neutral pH where Fe is in the form of insoluble Fe^3+^.

### Transcriptional analysis of iron-sulfur cluster assembly system

Nitrogenase is a complex [Fe-S] enzyme and the [Fe-S] clusters of nitrogenase play a critical function in electron transfer and in the reduction of substrates driven by the free energy liberated from Mg-ATP hydrolysis [3–5]. NifUS (*nifU* and *nifS* gene products), which mobilizes Fe and S for the assembly of small Fe/S fragments, were generally thought to be specialized for the assembly of the Fe_4_-S_4_ cluster of NifH. NifU and NifS are also involved in the assembly of the P-cluster and the FeMo-co of the NifDK component of nitrogenase [30]. *nifSU* are widely distributed in diazotrophs, such as *K. oxytoca* and *A. vinelandii.* In addition to *nifSU*, *isc* (*iscR*, *iscU*, *iscS*, *iscA*, *hscB, hscA*, *fdx*, and *iscX*)system also contributes the assembly of Fe-S cluster in *A. vinelandii* [31]*.*

**Fig. 4:**
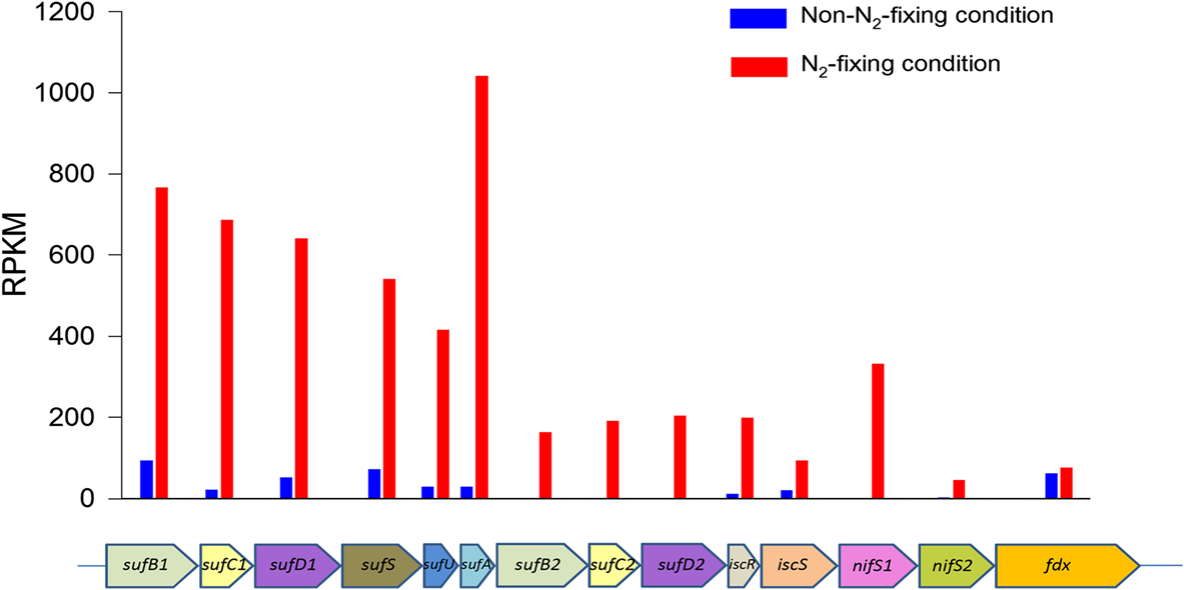
Differential expression of the genes involved in the synthesis of Fe-S cluster in N_2_-fixing and non-N_2_-fixing conditions

The genome of *Paenibacillus* sp. WLY78 does not have *nifSU*, but contains a complete *suf* (*sufCBSUD)* operon, a partial *suf* (*sufABC*) operon, a partial *isc* system (*iscSR* and *fdx*) and two *nifS*-like genes. This study reveals that the transcript abundances of *sufCBSUD* were much higher than the other related genes in N_2_-fixing condition, indicating that they play important roles in the Fe-S cluster assembly of nitrogenase (Fig. 4 and Additional file 1: Table S5). The expression of the partial *suf* (*sufABC*) operon, partial *isc* system (*iscSR* and *fdx*) and two *nifS*-like genes were also induced by N_2_-fixing condition.

### Transcriptional analysis of electron transporters for nitrogenase

Nitrogen fixation is carried out by the enzyme nitrogenase, which transfers electrons originating from low-potential electron carriers, such as flavodoxin or ferredoxin molecules, to molecular N_2_ [15]. In *K. oxytoca*, the physiological electron flow to nitrogenase involves specifically the products of the *nifF* and *nifJ* genes [17]. The *nifF* gene product, a flavodoxin, mediates electron transfer from the *nifJ* gene product, a pyruvate: flavodoxin oxidoreductase, to the Fe protein of nitrogenase [18].

**Fig. 5:**
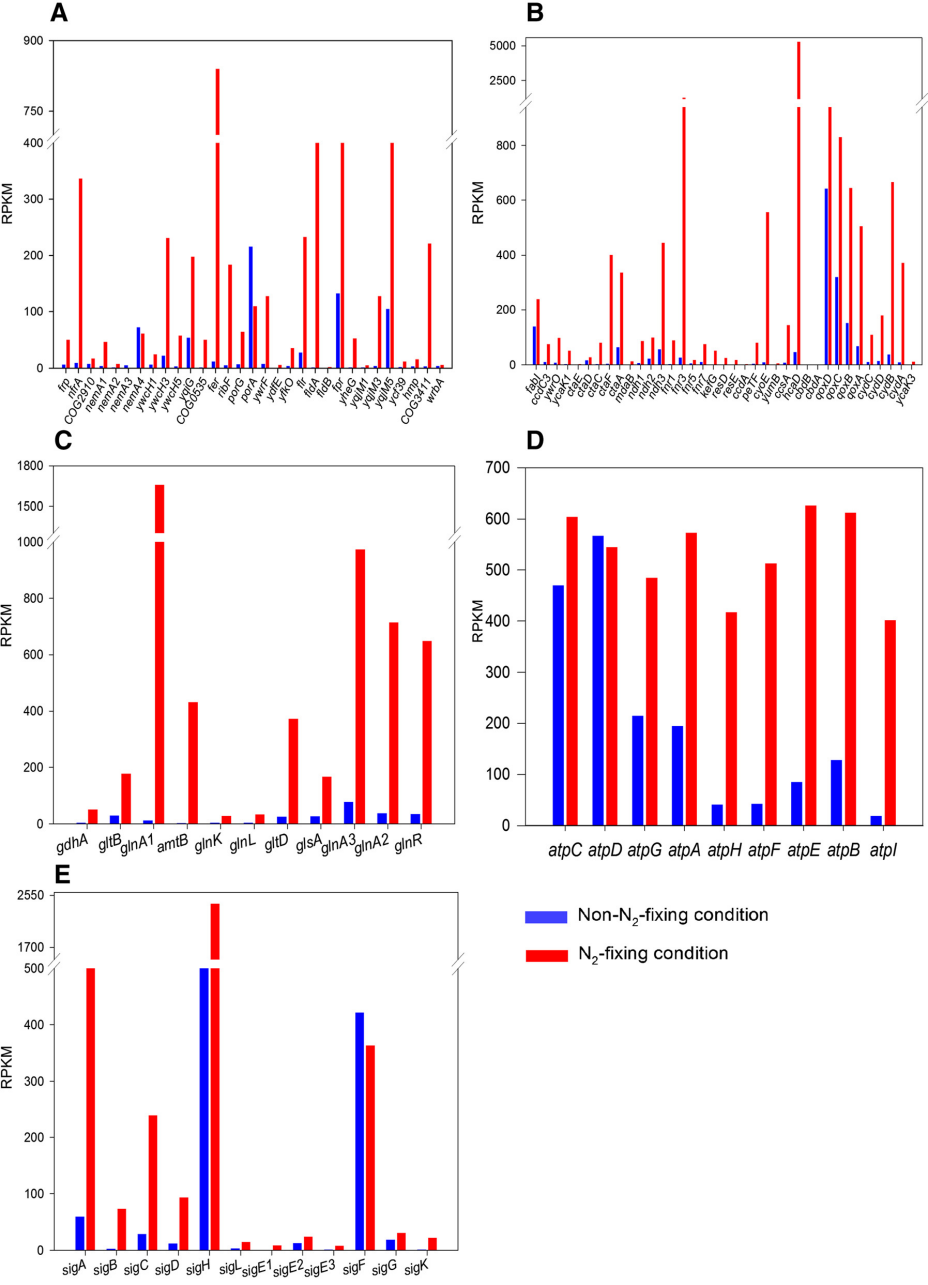
Differential expression of the genes relating to the electron transport, respiration and energy metabolism, nitrogen metabolism and ATP synthase in N_2_-fixing and non-N_2_-fixing conditions. **a** The genes specific for electron transport. **b** The genes involved in respiration and energy metabolism. **c** The genes specific for nitrogen metabolism. **d** The *atp* genes encoding ATP synthase. **e** The *sig* genes encoding sigma factor

Here, we find that there are several genes encoding ferredoxin, flavodoxin and flavodoxin oxidoreductase in the genome of *Paenibacillus* sp. WLY78. The *fer* encoding ferredoxin was the highest transcribed gene of these genes encoding ferredoxins in *Paenibacillus* sp. WLY78 in N_2_-fixing condition (Fig. 5a and Additional file 1: Table S6), suggesting that it may play an important role in electron transport. The *Paenibacillus fldA* encoding flavodoxin*,* showing 51 % identity with *A. vinelandii nifF* was up-regulated 310-fold in N_2_-fixing condition. But *fldB*, also encoding a ferredoxin, was transcribed at very low level in both conditions. *nfrA,* encoding NAD(P)H-flavin oxidoreductase, was up-regulated 34.2-fold in N_2_-fixing condition.

### Transcriptional analysis of respiration and energy metabolism

Since nitrogenase is very sensitive to oxygen, nitrogen fixation was carried out in anaerobic or microanaerobic conditions. From this study, it is found that the oxygen and nitrogen limitation induced about 20 cytochrome oxidase genes (*qoxABCD*, *ctaCDEF*, *cydAB* and others) in *Paenibacillus* sp. WLY78 (Fig. 5b and Additional file 1: Table S7). The *cydABCD* encoding cytochrome bd oxidase, active under microaerobic condition, were up-regulated from 12.2- to 42.35-fold. *ctaCDEF* were up-regulated from 1.56- to 88.73-fold. The *cyoABCD* coding for cytochrome bo oxidase, functional under aerobic condition, were weakly induced in N_2_-fixing condition. *qoxABCD* are up-regulated from 1.59- to 7.17-fold. There are 3 *ndh* genes encoding NADH dehydrogenase being up-regulated from 4.7- to 12.94-fold. *hcaD* encoding NAD(FAD)-dependent dehydrogenases was the highest up-regulated gene with 116.08-fold in N_2_-fixing condition compared to non-N_2_-fixing condition. The data suggest that active consumption of oxygen might provide O_2_ protection for nitrogenase in *Paenibacillus.*

Many bacteria are able to grow anaerobically using alternative electron acceptors, including nitrate or fumarate [32]. Here we found that there are two sets of *narIJHG* genes encoding assimilatory nitrite reductases and they were differently expressed from 0.37- to 4.98-fold in *Paenibacillus* (Additional file 1: Table S8). All of the *nas* genes (*nasE1B, nasE3D1, nasC* and *nasD3*) encoding NAD(P)H-nitrite-reductases and *nasA* encoding nitrate transporter were up-regulated in N_2_-fixing condition (Additional file 1: Table S8)*.* The two component regulatory proteins, ResD and ResE, and an anaerobic gene regulator, FNR, were previously shown to be indispensable for nitrate respiration in *B. subtilis* [33, 34]. Here we show that in *Paenibacillus* sp. WLY78, transcript level of *fnr* gene is up-regulated 6.84-fold, and *resD-resE* encoding two-component regulatory proteins ResD-ResE were up-regulated to 16.2- and 14.6-fold, respectively.

### Transcriptional analysis of nitrogen metabolism

The *nif* gene operon of *Paenibacillus* possesses a σ^70^-dependent promoter instead of a σ^54^-dependent promoter. GlnR, a global regulator of nitrogen metabolism in *Bacillus*, exists in *Paenibacillus* [35]. Our previous studies revealed that there is a GlnR/TnrA-binding site in the *nif* promoter region of *Paenibacillus* sp. WLY78 [35]. Here, we find that *Paenibacillus* sp. WLY78 has 3 *glnA* genes, one of which is linked with *glnR*, and the other two (here named *glnA1* and *glnA2*) are separately located in other regions of chromosome. The transcript level of *glnRA* operon was up-regulated 18.7- to 19.06-fold in N_2_-fixing condition compared to non-N_2_-fixing condition (Fig. 5c). The other 2 *glnA* genes, *amtB* and *gltABD* involved in nitrogen metabolism were significantly up-regulated in N_2_-fixing condition compared to non-N_2_-fixing condition (Fig. 5c and Additional file 1: Table S9).

### Transcriptional analysis of ATPase

The nitrogen fixation process is coupled to the hydrolysis of 16 equivalents of ATP. In *Paenibacillus* sp. WLY78, except for *atpD*, other *atp* genes (e.g. *atpC, atpG, atpA, atpH, atpF, atpE, atpB* and *atpI*) encoding ATP synthase subunits were highly expressed under N_2_-fixing condition compared to non-N_2_-fixing condition (Fig. 5d and Additional file 1: Table S10).

### Transcriptional analysis of the sigma factors

Regulation of gene expression in bacteria occurs primarily at the level of transcription. Although activators and repressors can significantly affect the efficiency of transcription, the specificity of the transcription reaction rests on interactions between RNA polymerase (RNAP) and the promoters [36]. The bacterial RNA polymerase holoenzyme (holo RNAP) is composed of core RNAP (α2β'βσω) and sigma (σ) factor. The σ factor of RNA polymerase recognizes promoter regions and initiates transcription [37].

It was reported that there are at least 10 known sigma factors in B. subtilis, including σ^A^, σ^B^, σ^C^, σ^D^, σ^E^, σ^F^, σ^G^, σ^H^, σ^K^, and σ^L^ [38]. The σ^A^ is the housekeeping sigma factor that is responsible for expression of essential genes [39]. Here we found that in *Paenibacillus* sp. WLY78, *sigA* gene encoding σ^A^ which is an equivalent of *E. coli* σ^70^, was up-regulated 10.72-fold in N_2_-fixing condition compared to non-N_2_-fixing condition (Fig. 5e and Additional file 1: Table S11). Moreover, *sigB* responsible for the transcription of genes that can confer stress resistance to the vegetative cell was up-regulated 29.64-fold, consistent with the current culture condition (limited nitrogen and oxygen). *sigH* specific for σ^H^ involved in chemotaxis/autolysin/flagellar gene transcription was the highest expressed gene among those genes specific for σ factors in both N_2_-fixing and non-N_2_-fixing conditions. *sigL* encoding σ^L^, which is an equivalent of σ^54^ of *E. coli* and responsible for nitrogen metabolism, was up-regulated in N_2_-fixing condition. *sigF* encoding σ^F^ specific for early forespore gene expression was highly expressed in non-N_2_-fixing condition and was down-regulated by N_2_-fixing condition. Other *sig* genes were transcribed at low levels in both N_2_-fixing and non-N_2_-fixing conditions.

## Discussion

In this study, a genome-wide transcription analysis of the nitrogen fixation in *Paenibacillus* sp. WLY78 cultured under N_2_-fixing and non-N_2_-fixing conditions was performed. Our results reveal that the transcripts of the *nif* genes (*nifBHDKENXhesAnifV*) of *Paenibacillus* sp. WLY78 are significantly up regulated in N_2_-fixing condition compared to non-N_2_-fixing condition, suggesting that *nif* gene expression in *Paenibacillus* was strongly regulated by ammonium and oxygen. Our data are consistent with the findings that in many diazotrophs such as *K. oxytoca* and *A. vinelandii*, expression of the *nif* genes is tightly controlled at the transcriptional level in response to the concentration of fixed nitrogen and the oxygen [18]. However, regulation mechanisms vary greatly among different diazotrophs. In the well-studied Gram-negative diazotrophic *K. oxytoc*a, *nif* genes, which possess a σ^54^-dependent promoter, are subject to two levels of regulation, one global and the other *nif* specific. The *nif*-specific regulation is mediated by the NifA (*nif* gene product) which is a transcriptional activator required for the expression of all *K. oxytoc*a *nif* operons, except its own [15]. The global level of *nif* regulation in *K. oxytoc*a is mediated by the global nitrogen regulator NtrC. The level of phosphorylated NtrC in the NtrB-NtrC two component regulatory system controls expression of *glnA-ntrBC* operon, *nifL-nifA* operon and *glnK-amtB* operon. The GlnD and the GlnB control the activity of the NtrB–NtrC two-component regulatory system [15].

Although regulation mechanism of nitrogen fixation is well-studied in Gram-negative bacteria, regulation mechanism of nitrogen fixation in Gram-positive *Paenibacillus* and *Bacillus* is lacking. There is no *nifA* in *Paenibacillus* sp. WLY78, and the *nif* gene operon of *Paenibacillus* possesses a σ^70^-dependent promoter instead of a σ^54^-dependent promoter. GlnR, a global regulator of nitrogen metabolism in *Bacillus*, exists in *Paenibacillus* [35]. Our previous studies revealed that there is a GlnR/TnrA-binding site in the *nif* promoter region of *Paenibacillus* sp. WLY78 [35]. Our recent studies reveals that GlnR binds the *nif* promoter in vitro by EMSA (Electrophoretic mobility shift assay) (not published), suggesting that GlnR might mediate *nif* gene transcription according to ammonium concentration.

Nitrogenase is a complex [Fe-S] enzyme. A lot of researches demonstrated that *nifU* and *nifS*, whose products were involved in the assembly of [Fe-S] clusters, were required for nitrogen fixation [30, 31]. The genome of *Paenibacillus* sp. WLY78 does not have *nifSU*, but contains a complete *suf* (*sufCBSUD)* operon, a partial *suf* (*sufABC*) operon, a partial *isc* system (*iscSR* and *fdx*) and two *nifS*-like genes. This study reveals that the transcript abundances of *sufCBSUD* were much higher than the other related genes in N_2_-fixing condition, indicating that they play important roles in the Fe-S cluster assembly of nitrogenase.

Nitrogen fixation is an energy intensive process and requires a suitable reductant to support electron transport to nitrogenase. Unlike *K. oxytoc*a *nif* gene cluster containing *nifF* and *nifJ*, which provide the electron transport, *Paenibacillus nif* gene cluster does not have *nifF* and *nifJ*. In this study, we found that several genes encoding ferredoxins, such as *fer*, *fldA* and *COG3411*, which might be involved in electron transport in nitrogenase of *Paenibacillus* sp. WLY78 were highly transcribed in N_2_-fixing condition.

## Conclusion

 In summary our results demonstrate that the expression of the *nif* gene operon of *Paenibacillus* was highly induced inN_2_-fixing condition. The non-*nif* genes specially required for nitrogen fixation, such as transporters of Fe, S and Mo were coordinately transcribed with *nif* genes in *Paenibacillus*. This study shows that Sul system was up regulated in N_2_-fixing condition, suggesting that Sul system, which takes place of *nifS* and *nifU*, plays important role in the synthesis of Fe-S cluster in *Paenibacillus*. We discover potential electron transporters which specifically transfer electrons to nitrogenase in *Paenibacillus*.

## Methods

### Bacterial strains, media and growth conditions

*Paenibacillus* sp. WLY78 used here was isolated from rhizosphere of bamboo by our laboratory (16). The bacterium was routinely grown in LB or LD medium (per liter contains: 2.5 g NaCl, 5 g yeast and 10 g tryptone) at 30°Cwith shaking. Since nitrogenase is very sensitive to oxygen and nitrogenase activity is inhibited by high concentration of ammonium, nitrogen fixation was carried out in anaerobic or microanaerobic condition and without ammonium or with limited ammonium. For transcriptomic analysis and real-time quantitative RT-PCR, *Paenibacillus* sp. WLY78 was grown in nitrogen-deficient medium under nitrogen-fixing condition (without O_2_ and NH_4_^+^) or non-nitrogen-fixing condition (21 % O_2_ and 100 mM NH_4_^+^). Nitrogen-deficient medium contained (per liter) (per liter) 10.4 g Na_2_HPO_4_, 3.4 g KH_2_PO_4_, 26 mg CaCl_2_• 2H_2_O, 30 mg MgSO_4_, 0.3 mg MnSO_4_, 36 mg Ferric citrate, 7.6 mg Na_2_MoO_4_ · 2H_2_O, 10 μg p-aminobenzoic acid, 5 μg biotin, 4 g glucose as carbon source and 2 mM glutamate as nitrogen source.

### Isolation of RNA

*Paenibacillus* sp. WLY78 was grown to OD_600_ = 0.3–0.4 at different concentration of ammonium and oxygen and then were harvested by centrifugation at 4 °C. Total RNA was isolated using a SV Total RNA Isolation System (Promega) according to the manufacturer’s instructions. The possibility of contamination of genomic DNA was eliminated by digestion with RNase-free DNase I (Takara Bio). The integrity and size distribution of the RNA was verified by agarose gel electrophoresis, and the concentration was determined spectrophotometrically.

### Transcriptomic analysis

Total RNA was isolated from *Paenibacillus* sp. WLY78 grown in N_2_-fixing and non-N_2_-fixing conditions, respectively. cDNA library construction and SOLiD sequencing from the total RNA were completed in Beijing Genomics Institute (Chinese Academy of Sciences) and three technical replicates of each sample were carried out. Raw sequencing reads were mapped against the *Paenibacillus* sp. WLY78 genome, using the programme BWA as previously described [40, 41]. We use DEGseq for identifying differentially expressed genes from RNA-seq data [42]. Transcript level differences with adjusted *P* values of <0.001 were considered to be significant.

### Quantitative real-time RT-PCR

To confirm the results of SOLiD sequencing, 9 *nif* genes (*nifBHDKENXhesAnifV*) were chosen for qRT-PCR analyses that were expressed in *Paenibacillus* sp. WLY78 from the two transcriptomes. qRT-PCR was performed using the SYBR GreenI(ROX) Kit from TakeRa Company according to the manufacturer’s protocol. Reactions were performed in triplicate. The primers used for qRT-PCR reactions are listed in Additional file 1: Table S12.

### Availability of data

The RNA-seq sequencing data of *Paenibacillus* sp. WLY78 have been deposited in NCBI database under accession number SRP053133.

## Additional files

Additional file 1
**Table S1.** Relative expression level (Reads Per Kilobase per million, RPKM) and fold changes (FC) in transcript levels of the *nif* genes in N_2_-fixing condition compared to non-N_2_-fixing condition in *Paenibacillus* sp.WLY78. **Table S2.** Relative expression level (RPKM) and fold changes (FC) in transcript levels of the *mod* genes in N_2_-fixing condition compared to non-N_2_-fixing condition in *Paenibacillus* sp.WLY78. **Table S3.** Relative expression level (RPKM) and fold changes (FC) in transcript levels of the sulfate transport related genes in N_2_-fixing condition compared to non-N_2_-fixing condition in *Paenibacillus* sp.WLY78. **Table S4.** Relative expression level (RPKM) and fold changes (FC) in transcript levels of the genes involved in iron transport, storage and regulation in N_2_-fixing condition compared to non-N_2_-fixing condition in *Paenibacillus* sp.WLY78. **Table S5.** Relative expression level (RPKM) and fold changes (FC) in transcript levels of the Fe-S cluster biosynthesis genes in N_2_-fixing condition compared to non-N_2_-fixing condition in *Paenibacillus* sp.WLY78. **Table S6.** Relative expression level (RPKM) and fold changes (FC) in transcript levels of the genes specific for electron transport in N_2_-fixing condition compared to non-N_2_-fixing condition in *Paenibacillus* sp.WLY78. **Table S7.** Relative expression level (RPKM) and fold changes (FC) in transcript levels of the genes specific for respiration and energy metabolism in N_2_-fixing condition compared to non-N_2_-fixing condition in *Paenibacillus* sp.WLY78. **Table S8.** Relative expression level (RPKM) and fold changes (FC) in transcript levels of the genes encoding nitrate/nitrite reductase in N_2_-fixing condition compared to non-N_2_-fixing condition in *Paenibacillus* sp.WLY78. **Table S9.** Relative expression level (RPKM) and fold changes (FC) in transcript levels of the genes specific for nitrogen metabolism in N_2_-fixing condition compared to non-N_2_-fixing condition in *Paenibacillus* sp.WLY78. **Table S10.** Relative expression level (RPKM) and fold changes (FC) in transcript levels of *atp* genes in N_2_-fixing condition compared to non-N_2_-fixing condition in *Paenibacillus* sp. WLY78. **Table S11.** Relative expression level (RPKM) and fold changes (FC) in transcript levels of the genes encoding sigma factor in N_2_-fixing condition compared to non-N_2_-fixing condition in *Paenibacillus* sp. WLY78. **Table S12.** Primers for qRT-PCR. (DOCX 64 kb)
