# Supplementary material for: Genome-wide transcriptome profiling of nitrogen fixation in Paenibacillus sp. WLY78
Source: BMC Microbiol. 2016 Mar 1;16:25. doi: 10.1186/s12866-016-0642-6 (PMC4774088; doi:10.1186/s12866-016-0642-6)
Supplement: Additional file 1 — Table S1. Relative expression level (Reads Per Kilobase per million, RPKM) and fold changes (FC) in transcript levels of the nif genes in N2-fixing condition compared to non-N2-fixing condition in Paenibacillus sp.WLY78. Table S2. Relative expression level (RPKM) and fold changes (FC) in transcript levels of the mod genes in N2-fixing condition compared to non-N2-fixing condition in Paenibacillus sp.WLY78. Table S3. Relative expression level (RPKM) and fold changes (FC) in transcript levels of the sulfate transport related genes in N2-fixing condition compared to non-N2-fixing condition in Paenibacillus sp.WLY78. Table S4. Relative expression level (RPKM) and fold changes (FC) in transcript levels of the genes involved in iron transport, storage and regulation in N2-fixing condition compared to non-N2-fixing condition in Paenibacillus sp.WLY78. Table S5. Relative expression level (RPKM) and fold changes (FC) in transcript levels of the Fe-S cluster biosynthesis genes in N2-fixing condition compared to non-N2-fixing condition in Paenibacillus sp.WLY78. Table S6. Relative expression level (RPKM) and fold changes (FC) in transcript levels of the genes specific for electron transport in N2-fixing condition compared to non-N2-fixing condition in Paenibacillus sp.WLY78. Table S7. Relative expression level (RPKM) and fold changes (FC) in transcript levels of the genes specific for respiration and energy metabolism in N2-fixing condition compared to non-N2-fixing condition in Paenibacillus sp.WLY78. Table S8. Relative expression level (RPKM) and fold changes (FC) in transcript levels of the genes encoding nitrate/nitrite reductase in N2-fixing condition compared to non-N2-fixing condition in Paenibacillus sp.WLY78. Table S9. Relative expression level (RPKM) and fold changes (FC) in transcript levels of the genes specific for nitrogen metabolism in N2-fixing condition compared to non-N2-fixing condition in Paenibacillus sp.WLY78. Table S10. Relative expression level (RPKM) a [file 12866_2016_642_MOESM1_ESM.docx]

**Genome-wide Transcriptome Profiling of Nitrogen Fixation in *Paenibacillus* sp. WLY78**

Haowen Shi, Liying Wang, Xinxin Li, Xiaomeng Liu，Tianyi Hao, Xiaojuan He and Sanfeng Chen*

Key Laboratory for Agrobiotechnology and Key laboratory of Soil Microbiology of Agriculture Ministry, China Agricultural University, Beijing 100193, P. R. China.

| **Table S1. Relative expression level (Reads Per Kilobase per million, RPKM) and fold changes (FC) in transcript levels of the *nif* genes in N_2_-fixing condition compared to non-N_2_-fixing condition in *Paenibacillus* sp.WLY78.** | | | | | | |
| --- | --- | --- | --- | --- | --- | --- |
|  | Locus Tag | RPKM  (Air + NH_4_^+^) | RPKM (Without O_2_ and NH_4_^+^) | FC | Gene name | Gene product |
| *Paenibacillus* sp. wly78 | S6001720 | 1.13 | 1174.51 | 1039.39 | *nifB* | Nitrogenase cofactor biosynthesis protein, NifB |
|  | S6001721 | 2.1 | 936.98 | 446.18 | *nifH* | Nitrogenase iron protein, NifH |
|  | S6001722 | 5.76 | 1769.99 | 307.29 | *nifD* | Nitrogenase molybdenum-iron protein alpha chains, NifD |
|  | S6001723 | 9.93 | 2140.29 | 215.54 | *nifK* | Nitrogenase molybdenum-iron protein beta chains, NifK |
|  | S6001724 | 10.06 | 2293.9 | 228.02 | *nifE* | Nitrogenase molybdenum-cofactor biosynthesis protein, NifE |
|  | S6001725 | 9.01 | 1432.07 | 158.94 | *nifN* | Nitrogenase molybdenum-cofactor biosynthesis protein, NifN |
|  | S6001726 | 5.66 | 1210.15 | 213.81 | *nifX* | Nitrogenase molybdenum-cofactor biosynthesis protein, NifX |
|  | S6001727 | 7.7 | 1820.22 | 236.39 | *hesA* | Molybdopterin/thiamine biosynthesis protein, HesA |
|  | S6001728 | 6.13 | 2284.18 | 372.62 | *nifV* | Nitrogen fixation homocitrate synthase, NifV |

| **Table S2. Relative expression level (RPKM) and fold changes (FC) in transcript levels of the *mod* genes in N_2_-fixing condition compared to non-N_2_-fixing condition in *Paenibacillus* sp.WLY78.** | | | | | | |
| --- | --- | --- | --- | --- | --- | --- |
|  | Locus Tag | RPKM  (Air + NH_4_^+^) | RPKM (Without O_2_ and NH_4_^+^) | FC | Gene name | Gene product |
| *Paenibacillus* sp. wly78 | S6000466 | 1.89 | 39.99 | 21.16 | *modF1* | ABC-type Mo transpoter, ATPase component/photorepair protein, ModF1 |
|  | S6000493 | 3.85 | 2.12 | 0.55 | *ModC* | ABC-type Mo transpoter, ATP-binding protein, ModC |
|  | S6000963 | 21.73 | 63.75 | 2.93 | *modB1* | ABC-type Mo transpoter, membrane protein, ModB1 |
|  | S6000964 | 11.95 | 73.73 | 6.17 | *modA1* | ABC-type Mo transpoter, periplasmicmolybdate-binding protein, ModA1 |
|  | S6001085 | 2.56 | 10.38 | 4.05 | *modB3* | ABC-type Mo transpoter, membrane protein, ModB3 |
|  | S6001086 | 3.78 | 8.95 | 2.37 | *modA3* | ABC-type Mo transpoter, periplasmicmolybdate-binding protein, ModA3 |
|  | S6003462 | 5.33 | 40.45 | 7.59 | *modF2* | ABC-type Mo transpoter, ATPase component/photorepair protein, ModF2 |
|  | S6004873 | 2.28 | 21.43 | 9.4 | *modB2* | ABC-type Mo transpoter, membrane protein, ModB2 |
|  | S6004874 | 2 | 25.11 | 12.56 | *modA2* | ABC-type Mo transpoter, periplasmicmolybdate-binding protein, ModA2 |
|  | S6004875 | 8.82 | 82.43 | 9.35 | *COG1910* | Periplasmicmolybdate-binding protein |

| **Table S3. Relative expression level (RPKM) and fold changes (FC) in transcript levels of the sulfate transport related genes in N_2_-fixing condition compared to non-N_2_-fixing condition in *Paenibacillus* sp.WLY78.** | | | | | | |
| --- | --- | --- | --- | --- | --- | --- |
|  | Locus Tag | RPKM  (Air + NH_4_^+^) | RPKM (Without O_2_ and NH_4_^+^) | FC | Gene name | Gene product |
| *Paenibacillus* sp. wly78 | S6002126 | 19.79 | 104.39 | 5.27 | *cysA* | ABC-type sulfate/molybdate transporters, ATPase component |
|  | S6003086 | 2.48 | 81.82 | 32.99 | *cysP* | Phosphate/sulphatepermeases |
|  | S6004884 | 17.98 | 48.19 | 2.68 | *sulP* | Sulfate permease and related transporters |
|  | S6005632 | 26.34 | 204.01 | 7.75 | *cysW* | ABC-type sulfate transporter, permease component |
|  | S6005633 | 17.12 | 89.87 | 5.25 | *cysT* | ABC-type sulfate transporter, permease component |
|  | S6005634 | 2.7 | 33.69 | 12.48 | *sbp* | ABC-type sulfate transporter, periplasmic component |
|  | S6000004 | 76.44 | 730.74 | 9.56 | *cysH* | Phosphoadenosinephosphosulfate reductase |
|  | S6000057 | 20.56 | 60.88 | 2.96 | *cysS* | Cysteinyl-tRNAsynthetase |
|  | S6000058 | 7.57 | 28.09 | 3.71 | *cysE* | Serine acetyltransferase |
|  | S6000096 | 431.54 | 568.19 | 1.32 | *cysI* | Sulfite reductase subunit beta |
|  | S6000218 | 88.94 | 628.97 | 7.07 | *cysK1* | Cysteine_synthase |
|  | S6000677 | 1.89 | 54.95 | 29.07 | *cysC* | Adenylylsulfate kinase |
|  | S6000978 | 23.36 | 5.73 | 0.25 | *cysJ* | Sulfite reductase subunit alpha |
|  | S6003693 | 4.73 | 28.67 | 6.06 | *cysK3* | Cysteine synthase |
|  | S6003805 | 10.35 | 87.53 | 8.46 | *cysK5* | Cysteine synthase |
|  | S6004670 | 5.55 | 113.37 | 20.43 | *cysG* | Siroheme synthase |
|  | S6005496 | 39.43 | 176.23 | 4.47 | *cysK7* | Cysteine synthase |
|  | S6001238 | 24.03 | 62.59 | 2.6 | *sul1* | Sulfate permease and related transporters |

| **Table S4. Relative expression level (RPKM) and fold changes (FC) in transcript levels of the genes involved in iron transport, storage and regulation in N_2_-fixing condition compared to non-N_2_-fixing condition in *Paenibacillus* sp.WLY78.** | | | | | | |
| --- | --- | --- | --- | --- | --- | --- |
|  | Locus Tag | RPKM  (Air + NH_4_^+^) | RPKM (Without O_2_ and NH_4_^+^) | FC | Gene name | Gene product |
| *Paenibacillus* sp. wly78 | S6000494 | 2.84 | 2.87 | 1.01 | *afuA* | ABC-type Fe^3+^ transporter, periplasmic component |
|  | S6000589 | 30.98 | 497.78 | 16.07 | *ftn* | Ferritin protein |
|  | S6003097 | 1.63 | 35.66 | 21.88 | *fhuB3* | ABC-type Fe^3+^-siderophore transporter, permease component |
|  | S6002343 | 2.04 | 156.36 | 76.65 | *fhuB5* | ABC-type Fe^3+^-siderophore transporter, permease component |
|  | S6000649 | 3.59 | 79.04 | 22.02 | *fhuB7* | ABC-type Fe^3+^-siderophore transporter, permease component |
|  | S6003538 | 0.83 | 62.82 | 75.69 | *fhuB9* | ABC-type Fe^3+^-siderophore transporter, permease component |
|  | S6003124 | 5.84 | 35.66 | 6.11 | *fhuB11* | ABC-type Fe^3+^-siderophore transporter, permease component |
|  | S6003125 | 5.16 | 33.13 | 6.42 | *fhuC1* | ABC-type cobalamin/Fe^3+^-siderophores transporters, ATPase component |
|  | S6005550 | 1.3 | 72.49 | 55.76 | *fhuC3* | ABC-type cobalamin/Fe^3+^-siderophores transporters, ATPase component |
|  | S6004847 | 39.38 | 566.45 | 14.38 | *fhuC5* | ABC-type cobalamin/Fe^3+-^siderophores transporters, ATPase component |
|  | S6003123 | 1.53 | 32.56 | 21.28 | *fhuD1* | ABC-type Fe^3+^-hydroxamate transporter, periplasmic component |
|  | S6000650 | 1.25 | 103.26 | 82.61 | *fhuD3* | ABC-type Fe^3+^-hydroxamate transporter, periplasmic component |
|  | S6003235 | 2.99 | 614.83 | 205.63 | *fhuD7* | ABC-type Fe^3+^-hydroxamate transporter, periplasmic component |
|  | S6001123 | 1.7 | 63.41 | 37.3 | *fhuD9* | ABC-type Fe^3+^-hydroxamate transporter, periplasmic component |
|  | S6005548 | 2.1 | 125.76 | 59.89 | *fhuD11* | ABC-type Fe^3+^-hydroxamate transporter, periplasmic component |
|  | S6003096 | 1.01 | 22.85 | 22.62 | *fhuG1* | ABC-type Fe^3+^-siderophore transporter, permease component |
|  | S6000648 | 3.17 | 55.49 | 17.5 | *fhuG3* | ABC-type Fe^3+^-siderophore transporter, permease component |
|  | S6003539 | 1.08 | 59.92 | 55.48 | *fhuG5* | ABC-type Fe^3+^-siderophore transporter, permease component |
|  | S6005549 | 2.64 | 66.22 | 25.08 | *fhuG7* | ABC-type Fe^3+^-siderophore transporter, permease component |
|  | S6001013 | 2.67 | 167.92 | 62.89 | *yfiY* | ABC Fe^3+^transporter, permease component |
|  | S6001015 | 1.08 | 14.77 | 13.68 | *fecB* | Iron(III) dicitrate transporter permease protein, FecB |
|  | S6002136 | 3.52 | 18.21 | 5.17 | *fecD1* | ABC-type Fe^3+^-siderophore transporter, permease component |
|  | S6002176 | 1.13 | 134.42 | 118.96 | *fecD3* | ABC-type Fe3+-siderophore transporter, permease component |
|  | S6005517 | 10.57 | 68.53 | 6.48 | *fecD5* | ABC-type Fe^3+^-siderophore transporter, permease component |
|  | S6005518 | 4.85 | 35.9 | 7.4 | *fecE* | ABC-type cobalamin/Fe^3+^-siderophores transporters, ATPase component |
|  | S6002069 | 10.24 | 41.86 | 4.09 | *ybbB3* | ABC-type Fe^3+^-hydroxamate transporter, periplasmic component |
|  | S6003717 | 1.57 | 143.47 | 91.38 | *yxeB3* | ABC-type Fe^3+^-hydroxamate transporter, periplasmic component |
|  | S6002070 | 1.6 | 54.04 | 33.78 | *yxeB5* | ABC-type Fe^3+^-hydroxamate transporter, periplasmic component |
|  | S6002451 | 1.67 | 39.07 | 23.4 | *yxeB7* | ABC-type Fe^3+^-hydroxamate transporter, periplasmic component |
|  | S6003537 | 1.17 | 49.95 | 42.69 | *yxeB9* | ABC-type Fe^3+^-hydroxamate transporter, periplasmic component |
|  | S6003535 | 0.72 | 45.14 | 62.69 | *yxeB11* | ABC-type Fe^3+^-hydroxamate transporter, periplasmic component |
|  | S6002135 | 2.56 | 19.13 | 7.47 | *yurC* | ABC-type Fe^3+^-hydroxamate transporter, periplasmic component |
|  | S6002137 | 3.12 | 16.77 | 5.38 | *fepC* | ABC-type cobalamin/Fe^3+^-siderophores transporters, ATPase component |
|  | S6002175 | 1.51 | 169.42 | 112.2 | *feuA* | ABC-type Fe^3+^-hydroxamate transporter, periplasmic component |
|  | S6002177 | 1.1 | 69.19 | 62.9 | *feuC* | ABC-type Fe^3+^-siderophore transporter, permease component |
|  | S6002510 | 60.67 | 204.97 | 3.38 | *hemH* | Protohemeferro-lyase (ferrochelatase) |
|  | S6003111 | 32.08 | 123.26 | 3.84 | *cbrA1* | ABC-type Fe^3+^-citrate transporter, periplasmic component |
|  | S6003095 | 1.23 | 31.35 | 25.49 | *cbrA3* | ABC-type Fe^3+^-hydroxamate transporter, periplasmic component |
|  | S6002342 | 1.28 | 188.49 | 147.26 | *cbrA5* | ABC-type Fe^3+^-hydroxamate transporter, periplasmic component |
|  | S6003112 | 45.18 | 20.56 | 0.46 | *yfmD* | ABC-type Fe^3+^-siderophore transporter, permease component |
|  | S6003114 | 41.47 | 27.87 | 0.67 | *yfmE* | Iron (III) dicitrate ABC transporter permease |
|  | S6003169 | 2.15 | 234.18 | 108.92 | *perR1* | Fe^2+^/Zn^2+^ uptake regulation proteins |
|  | S6004921 | 21.32 | 957.77 | 44.92 | *perC3* | Fe^2+^/Zn^2+^ uptake regulation proteins |
|  | S6003225 | 15.08 | 177.62 | 11.78 | *fur3* | Fe^2+^/Zn^2+^ uptake regulation proteins |
|  | S6003285 | 7.89 | 346.22 | 43.88 | *fur1* | Fe^2+^/Zn^2+^ uptake regulation proteins |
|  | S6005519 | 5.9 | 22.34 | 3.79 | *hmuT* | ABC-type Fe^3+^-hydroxamate transporter, periplasmic component |
|  | S6005562 | 3.75 | 204.48 | 54.53 | *feoA* | Ferrous iron transporter protein A |
|  | S6005563 | 24.13 | 290.34 | 12.03 | *feoB* | Ferrous iron transporter protein B |

| **Table S5. Relative expression level (RPKM) and fold changes (FC) in transcript levels of the Fe-S cluster biosynthesis genes in N_2_-fixing condition compared to non-N_2_-fixing condition in *Paenibacillus* sp.WLY78.** | | | | | | |
| --- | --- | --- | --- | --- | --- | --- |
|  | Locus Tag | RPKM  (Air + NH_4_^+^) | RPKM (Without O_2_ and NH_4_^+^) | FC | Gene name | Gene product |
| *Paenibacillus* sp. wly78 | S6000450 | 2.47 | 332.75 | 134.72 | *nifS1*-like | Cysteine desulfurase |
|  | S6003911 | 3.33 | 44.74 | 13.44 | *nifS2*-like | Cysteine desulfurase |
|  | S6001335 | 19.92 | 93.76 | 4.71 | *iscS* | Cysteine desulfurase |
|  | S6001336 | 10.76 | 198.33 | 18.43 | *iscR* | DNA-binding transcriptional regulator, IscR |
|  | S6002250 | 61.17 | 75.83 | 1.24 | *fdx* | Ferredoxin protein |
|  | S6002382 | 20.59 | 686.58 | 33.35 | *sufC1* | Fe-S assembly ATPase, SufC |
|  | S6002383 | 51.24 | 641.21 | 12.51 | *sufD1* | Fe-S cluster assembly protein, SufD |
|  | S6002384 | 72.36 | 540.34 | 7.47 | *sufS* | Cysteine desulfurase |
|  | S6002385 | 27.86 | 416.69 | 14.96 | *sufU* | Fe-S cluster assembly protein, SufU |
|  | S6002386 | 93.66 | 766.97 | 8.19 | *sufB1* | Fe-S cluster assembly protein, SufB |
|  | S6004343 | 1.9 | 190.89 | 100.47 | *sufC2* | Fe-S assembly ATPase, SufC |
|  | S6004344 | 1.87 | 204.14 | 109.17 | *sufD2* | Fe-S cluster assembly protein, SufD |
|  | S6004345 | 2.18 | 162.96 | 74.75 | *sufB2* | Fe-S cluster assembly protein, SufB |
|  | S6004436 | 28.28 | 1041.19 | 36.82 | *sufA* | Fe-S cluster assembly accessory protein, SufA |

| **Table S6. Relative expression level (RPKM) and fold changes (FC) in transcript levels of the genes specific for electron transport in N_2_-fixing condition compared to non-N_2_-fixing condition in *Paenibacillus* sp.WLY78.** | | | | | | |
| --- | --- | --- | --- | --- | --- | --- |
|  | Locus Tag | RPKM  (Air + NH_4_^+^) | RPKM (Without O_2_ and NH_4_^+^) | FC | Gene name | Gene product |
| *Paenibacillus* sp. wly78 | S6000275 | 6.4 | 49.8 | 7.78 | *frp* | NAD(P)H-flavinoxidoreductase |
|  | S6000287 | 9.78 | 336.59 | 34.42 | *nfrA* | NAD(P)H-flavinoxidoreductase |
|  | S6000342 | 7.37 | 16.58 | 2.25 | *COG2910* | NADH-flavin reductase |
|  | S6000364 | 3.89 | 46.03 | 11.83 | *nemA1* | NADH:flavinoxidoreductases, Old Yellow Enzyme family |
|  | S6000653 | 1.19 | 7.9 | 6.64 | *nemA2* | NADH:flavinoxidoreductases, Old Yellow Enzyme family |
|  | S6000710 | 5.07 | 0.7 | 0.14 | *nemA3* | NADH:flavinoxidoreductases, Old Yellow Enzyme family |
|  | S6005579 | 71.88 | 60.87 | 0.85 | *nemA4* | NADH:flavinoxidoreductases, Old Yellow Enzyme family |
|  | S6001866 | 6.24 | 24.34 | 3.9 | *ywcH1* | Flavin-dependent oxidoreductases |
|  | S6001250 | 21.64 | 231.36 | 10.69 | *ywcH3* | Flavin-dependent oxidoreductases |
|  | S6004848 | 3.37 | 57.48 | 17.06 | *ywcH5* | Flavin-dependent oxidoreductases |
|  | S6001639 | 53.5 | 198.12 | 3.7 | *yqiG* | NADH:flavinoxidoreductases, Old Yellow Enzyme family |
|  | S6002448 | 1.4 | 49.68 | 35.49 | *COG0535* | Fe-S oxidoreductase |
|  | S6002547 | 12.12 | 839.59 | 69.27 | *fer* | Ferredoxin |
|  | S6002947 | 5.33 | 183.81 | 34.49 | *ribF* | Riboflavin biosynthesis protein, ribF |
|  | S6003300 | 7.17 | 64.32 | 8.97 | *porG* | Pyruvate:ferredoxinoxidoreductase, gamma subunit |
|  | S6003301 | 216.01 | 109.87 | 0.51 | *porA* | Pyruvate:ferredoxinoxidoreductase, alpha subunit |
|  | S6003404 | 7.38 | 127.53 | 17.28 | *ywrF* | Flavin reductase-like protein, YwrF |
|  | S6003586 | 0.42 | 5.79 | 13.79 | *ydfE* | Flavoproteinoxygenases |
|  | S6003719 | 4.22 | 35.35 | 8.38 | *yfkO* | NAD(P)H-flavinoxidoreductase |
|  | S6003919 | 27.02 | 233.07 | 8.63 | *flr* | Flavoredoxin |
|  | S6004317 | 1.6 | 496.41 | 310.26 | *fldA* | Flavodoxin 1 |
|  | S6005176 | 0.98 | 1.82 | 1.86 | *fldB* | Flavodoxin 2 |
|  | S6004456 | 132.44 | 449.96 | 3.4 | *fpr* | Ferredoxin-NADP reductase |
|  | S6004601 | 1.32 | 52.4 | 39.7 | *yheG* | Flavin reductase |
|  | S6004740 | 1.3 | 5.29 | 4.07 | *yqjM1* | NADH:flavinoxidoreductases, Old Yellow Enzyme family |
|  | S6003510 | 3.66 | 127.7 | 34.89 | *yqjM3* | NADH:flavinoxidoreductases, Old Yellow Enzyme family |
|  | S6005093 | 104.74 | 472.64 | 4.51 | *yqjM5* | NADH:flavinoxidoreductases, Old Yellow Enzyme family |
|  | S6004986 | 2.24 | 12.04 | 5.38 | *ycf39* | NADH-flavin reductase |
|  | S6005113 | 3.47 | 15.46 | 4.46 | *hmp* | Flavodoxin reductases (ferredoxin-NADPH reductases) family 1 |
|  | S6005408 | 3.42 | 221.26 | 64.7 | *COG3411* | Ferredoxin |
|  | S6005572 | 3.62 | 5.71 | 1.58 | *wrbA* | Multimericflavodoxin, WrbA |

| **Table S7. Relative expression level (RPKM) and fold changes (FC) in transcript levels of the genes specific for respiration and energy metabolism in N_2_-fixing condition compared to non-N_2_-fixing condition in *Paenibacillus* sp.WLY78.** | | | | | | |
| --- | --- | --- | --- | --- | --- | --- |
|  | Locus Tag | RPKM  (Air + NH_4_^+^) | RPKM (Without O_2_ and NH_4_^+^) | FC | Gene name | Gene product |
| *Paenibacillus* sp. wly78 | S6000231 | 138.63 | 239.39 | 1.73 | *fabI* | Enoyl-[acyl-carrier-protein] reductase (NADH) |
|  | S6000332 | 10.25 | 74.56 | 7.27 | *ccdC3* | Membrane protein involved in cytochrome C biogenesis |
|  | S6000596 | 7.95 | 96.67 | 12.16 | *ywrO* | NADPH-quinone reductase (modulator of drug activity B) |
|  | S6000647 | 2.05 | 50.2 | 24.49 | *ycaK1* | NADPH-quinone reductase (modulator of drug activity B) |
|  | S6000788 | 0.38 | 2.06 | 5.42 | *ctaE* | Cytochrome C oxidase subunit III |
|  | S6001682 | 16.82 | 26.21 | 1.56 | *ctaD* | Cytochrome C oxidase subunit I |
|  | S6002247 | 3.19 | 79.61 | 24.96 | *ctaC* | Cytochrome C oxidase subunit II |
|  | S6002690 | 4.53 | 400.32 | 88.37 | *ctaF* | Cytochrome C oxidase subunit IV |
|  | S6004423 | 63.72 | 335.79 | 5.27 | *ctaA* | Cytochrome oxidase assembly |
|  | S6001217 | 1.25 | 13.01 | 10.41 | *mdaB* | NADPH-quinone reductase (modulator of drug activity B) |
|  | S6001653 | 6.63 | 85.82 | 12.94 | *ndh1* | NADH dehydrogenase, FAD-containing subunit |
|  | S6004433 | 21.01 | 98.74 | 4.7 | *ndh2* | NADH dehydrogenase, FAD-containing subunit |
|  | S6005032 | 55.79 | 445.4 | 7.98 | *ndh3* | NADH dehydrogenase, FAD-containing subunit |
|  | S6001676 | 2.45 | 88.61 | 36.17 | *fnr1* | Anaerobic regulatory protein |
|  | S6003218 | 25.4 | 1262.28 | 49.7 | *fnr3* | Anaerobic regulatory protein |
|  | S6004820 | 5.15 | 18.54 | 3.6 | *fnr5* | Anaerobic regulatory protein |
|  | S6005182 | 10.88 | 74.39 | 6.84 | *fnr7* | Anaerobic regulatory protein |
|  | S6002482 | 1.65 | 50.88 | 30.84 | *kefG* | NADPH-quinone reductase (modulator of drug activity B) |
|  | S6002756 | 1.5 | 24.32 | 16.21 | *resD* | Transcriptional regulatory protein, ResD |
|  | S6002757 | 1.21 | 17.69 | 14.62 | *resE* | Two-component sensor histidine kinase |
|  | S6003228 | 1.3 | 3.26 | 2.51 | *ccdA* | Cytochrome c biogenesis protein |
|  | S6003337 | 3.41 | 79.25 | 23.24 | *peTF* | Na+-transporting NADH:ubiquinoneoxidoreductase |
|  | S6004411 | 8.42 | 556.8 | 66.13 | *cyoE* | Polyprenyltransferase, cytochrome oxidase assembly factor |
|  | S6004457 | 1.15 | 5.36 | 4.66 | *yumB* | NADH dehydrogenase, FAD-containing subunit |
|  | S6004669 | 8.12 | 144.6 | 17.81 | *ccsA* | Cytochrome c biogenesis protein |
|  | S6004843 | 45.64 | 5298 | 116.08 | *hcaD* | NAD(FAD)-dependent dehydrogenases |
|  | S6005554 | 1.47 | 1.69 | 1.15 | *cbdB* | Cytochrome bd-type quinol oxidase, subunit 2 |
|  | S6005555 | 1.35 | 1.44 | 1.07 | *cbdA* | Cytochrome bd-type quinol oxidase, subunit 1 |
|  | S6005580 | 641.46 | 1018.29 | 1.59 | *qoxD* | Heme/copper-type cytochrome/quinol oxidase, subunit 4 |
|  | S6005581 | 319.81 | 830.17 | 2.6 | *qoxC* | Heme/copper-type cytochrome/quinol oxidase, subunit 3 |
|  | S6005582 | 152.01 | 645 | 4.24 | *qoxB* | Heme/copper-type cytochrome/quinol oxidases, subunit 1 |
|  | S6005583 | 66.86 | 505.89 | 7.57 | *qoxA* | Heme/copper-type cytochrome/quinol oxidases, subunit 2 |
|  | S6005593 | 10.37 | 108.25 | 10.44 | *cydC* | ABC-type transportr involved in cytochrome bd biosynthesis |
|  | S6005594 | 14.66 | 179.76 | 12.26 | *cydD* | ABC-type transportr involved in cytochrome bd biosynthesis |
|  | S6005595 | 36.04 | 666.04 | 18.48 | *cydB* | Cytochrome bd-type quinol oxidase, subunit 2 |
|  | S6005596 | 8.79 | 372.29 | 42.35 | *cydA* | Cytochrome bd-type quinol oxidase, subunit 1 |
|  | S6005660 | 1.14 | 12.07 | 10.59 | *ycaK3* | NADPH-quinone reductase (modulator of drug activity B) |

| **Table S8. Relative expression level (RPKM) and fold changes (FC) in transcript levels of the genes encoding nitrate/nitrite reductase in N_2_-fixing condition compared to non-N_2_-fixing condition in *Paenibacillus* sp.WLY78.** | | | | | | |
| --- | --- | --- | --- | --- | --- | --- |
|  | Locus Tag | RPKM  (Air + NH_4_^+^) | RPKM (Without O_2_ and NH_4_^+^) | FC | Gene name | Gene product |
| *Paenibacillus* sp. wly78 | S6000096 | 431.54 | 568.19 | 1.32 | *nirA* | Ferredoxin-nitrite reductase |
|  | S6005057 | 1.23 | 3.83 | 3.11 | *firD* | Anaerobic nitric oxide reductase flavorubredoxin |
|  | S6005058 | 1.93 | 5.82 | 3.02 | *nirB* | Nitric oxidereductaseflavoprotein |
|  | S6000812 | 13.36 | 58.75 | 4.4 | *narI1* | Nitrate reductase gamma subunit |
|  | S6000813 | 4.94 | 24.59 | 4.98 | *narJ1* | Nitrate reductase delta subunit |
|  | S6000814 | 6.39 | 7.57 | 1.18 | *narH1* | Nitrate reductase beta subunit |
|  | S6000815 | 5.79 | 2.36 | 0.41 | *narG1* | Nitrate reductase alpha subunit |
|  | S6000819 | 2.22 | 0.76 | 0.34 | *narK1* | Nitrate/nitrite transporter |
|  | S6001076 | 1.74 | 3.44 | 1.98 | *narK2* | Nitrate/nitrite transporter |
|  | S6001081 | 1.93 | 6.63 | 3.44 | *narI2* | Nitrate reductase gamma subunit |
|  | S6001082 | 3.9 | 2.87 | 0.74 | *narJ2* | Nitrate reductase delta subunit |
|  | S6001083 | 5.53 | 2.68 | 0.48 | *narH2* | Nitrate reductase beta subunit |
|  | S6001084 | 4.85 | 1.8 | 0.37 | *narG2* | Nitrate reductase alpha subunit |
|  | S6001513 | 47.61 | 123.18 | 2.59 | *nasE1* | Assimilatory nitrite reductase |
|  | S6001514 | 20.75 | 74.59 | 3.59 | *nasB* | Nitrite reductase large subunit |
|  | S6003771 | 1.83 | 154.31 | 84.32 | *nasE3* | Assimilatory nitrite reductase |
|  | S6003772 | 0.91 | 157.59 | 173.18 | *nasD1* | NAD(P)H-nitrite reductase |
|  | S6003778 | 1.28 | 39.93 | 31.2 | *nasC* | Assimilatory nitrate reductase catalytic subunit |
|  | S6003781 | 1.06 | 19.81 | 18.69 | *nasD3* | NAD(P)H-nitrite reductase |
|  | S6003782 | 3.21 | 93.63 | 29.17 | *nasA* | Nitrate transporter |

| **Table S9.** **Relative expression level (RPKM) and fold changes (FC) in transcript levels of the genes specific for nitrogen metabolism in N_2_-fixing condition compared to non-N_2_-fixing condition in *Paenibacillus* sp.WLY78.** | | | | | | |
| --- | --- | --- | --- | --- | --- | --- |
|  | Locus Tag | RPKM  (Air + NH_4_^+^) | RPKM (Without O_2_ and NH_4_^+^) | FC | Gene name | Gene product |
| *Paenibacillus* sp. wly78 | S6001131 | 4.21 | 51.44 | 12.22 | *gdhA* | Glutamate dehydrogenase/leucine dehydroGenase |
|  | S6001872 | 29.74 | 177.68 | 5.97 | *gltB* | Glutamate synthase domain 2 |
|  | S6002289 | 12.4 | 1659.32 | 133.82 | *glnA1* | Glutamine synthetase |
|  | S6002541 | 2.15 | 431.01 | 200.47 | *amtB* | Ammonia permease |
|  | S6002773 | 4.4 | 27.84 | 6.33 | *glnK* | Signal transduction histidine kinase |
|  | S6002774 | 4.17 | 33.86 | 8.12 | *glnL* | Sensory transduction protein |
|  | S6003492 | 25.35 | 373.18 | 14.72 | *gltD* | NADPH-dependent glutamate synthase beta chain |
|  | S6003575 | 27.45 | 167.66 | 6.11 | *glsA* | Glutaminase |
|  | S6004014 | 77.42 | 974.52 | 12.59 | *glnA3* | Glutamine synthetase |
|  | S6004043 | 37.48 | 714.38 | 19.06 | *glnA2* | Glutamine synthetase |
|  | S6004044 | 34.63 | 647.41 | 18.7 | *glnR* | Predicted transcriptional regulators |

| **Table S10. Relative expression level (RPKM) and fold changes (FC) in transcript levels of *atp* genes in N_2_-fixing condition compared to non-N_2_-fixing condition in *Paenibacillus* sp. WLY78.** | | | | | | |
| --- | --- | --- | --- | --- | --- | --- |
|  | Locus Tag | RPKM  (Air + NH_4_^+^) | RPKM (Without O_2_ and NH_4_^+^) | FC | Gene name | Gene product |
| *Paenibacillus* sp. wly78 | S6004789 | 469.97 | 603.78 | 1.28 | *atpC* | F0F1-type ATP synthase, epsilon subunit |
|  | S6004790 | 567.08 | 544.48 | 0.96 | *atpD* | F0F1-type ATP synthase, beta subunit |
|  | S6004791 | 214.55 | 484.95 | 2.26 | *atpG* | F0F1-type ATP synthase, gamma subunit |
|  | S6004792 | 194.47 | 572.68 | 2.94 | *atpA* | F0F1-type ATP synthase, alpha subunit |
|  | S6004793 | 41.27 | 417.09 | 10.11 | *atpH* | F0F1-type ATP synthase, delta subunit |
|  | S6004794 | 43.31 | 513.05 | 11.85 | *atpF* | F0F1-type ATP synthase, subunit B |
|  | S6004795 | 84.88 | 626.1 | 7.38 | *atpE* | F0F1-type ATP synthase, subunit C |
|  | S6004796 | 128.36 | 612.17 | 4.77 | *atpB* | F0F1-type ATP synthase, subunit A |
|  | S6004797 | 19.34 | 401.45 | 20.76 | *atpI* | F0F2-type ATP synthase, subunit I |

| **Table S11. Relative expression level (RPKM) and fold changes (FC) in transcript levels of the genes encoding sigma factor in N_2_-fixing condition compared to non-N_2_-fixing condition in *Paenibacillus* sp.WLY78.** | | | | | | |
| --- | --- | --- | --- | --- | --- | --- |
|  | Locus Tag | RPKM  (Air + NH_4_^+^) | RPKM (Without O_2_ and NH_4_^+^) | FC | Gene name | Gene product |
| *Paenibacillus* sp. wly78 | S6003923 | 59.7 | 640.01 | 10.72 | *sigA* | RNA polymerase sigma factor, SigA |
|  | S6001066 | 2.48 | 73.51 | 29.64 | *sigB* | RNA polymerase sigma factor, SigB |
|  | S6000905 | 28.97 | 239.15 | 8.26 | *sigC* | RNA polymerase sigma factor, SigC |
|  | S6002923 | 12.3 | 93.43 | 7.6 | *sigD* | RNA polymerase sigma factor, SigD |
|  | S6000053 | 522 | 2413.38 | 4.62 | *sigH* | RNA polymerase sigma factor, SigH |
|  | S6000310 | 3.28 | 14.97 | 4.56 | *sigL* | RNA polymerase sigma factor, SigL |
|  | S6000108 | 0.94 | 8.67 | 9.22 | *sigE1* | RNA polymerase sigma-E factor |
|  | S6003876 | 13.08 | 24.26 | 1.85 | *sigE2* | RNA polymerase sigma-E factor |
|  | S6003025 | 1.45 | 8.05 | 5.55 | *sigE3* | RNA polymerase sigma-E factor |
|  | S6003294 | 421.8 | 363.33 | 0.86 | *sigF* | RNA polymerase sigma factor, SigF |
|  | S6003875 | 18.69 | 31.23 | 1.67 | *sigG* | RNA polymerase sigma factor, SigG |
|  | S6000947 | 1.61 | 21.77 | 13.52 | *sigK* | RNA polymerase sigma factor, SigK |

**Table S12. Primers for qRT-PCR**

| Gene name | Forward Primer (5' - 3') | Reverse Primer (5' - 3') | Location /Target |
| --- | --- | --- | --- |
| *nifB* | *nifB*-F (GGATAAGCCTGAAGCGAGC | *nifB*-R (CAATCGGCTTTCCCGTGAC) | qRT-PCR |
| *nifH* | *nifH*-F (AACAGCCGGAATACGGACC) | *nifH*-R (ACCTGCCAGCTCTTCATACTC) | qRT-PCR |
| *nifD* | *nifD*-F (TCATTCCTGTACGCTGTGAGG) | *nifD*-R (CACCGCCGATATTGTAGTCTC) | qRT-PCR |
| *nifK* | *nifK*-F(GCGGAGATGATTGCGGTATG) | *nifK*-R (GGCGTCATAGCCTGTAATATGTG) | qRT-PCR |
| *nifE* | *nifE*-F (TCCCGCTGTGTTTGTCTATACC) | *nifE*-R (GTGCTGCCGATAATATGCTGG) | qRT-PCR |
| *nifN* | *nifN*-F (AGCTACTAATGGATGCCGTACTC) | *nifN*-R (CCGGACAAGGAAGTGGAAATATC) | qRT-PCR |
| *nifX* | *nifX*-F (CTGGGGAGCGATGAGAATGAG) | *nifX*-R (TCCTCAATGGTGCTACCGAAG) | qRT-PCR |
| *hesA* | *ORF*-F (GGATGGAGACGGCATTACAG) | *ORF*-R (GTTCAGCGCATATCTTTCCGG) | qRT-PCR |
| *nifV* | *nifV*-F (GCCATAGCTGCCCGTATAGA) | *nifV*-R (CTCCAGCGCGGTATTACCTG) | qRT-PCR |
| 16S rDNA | 16S-F (TTTGTCGTCAGCTCGTGTTCGTG) | 16S-R (ATCCCCACCTTCCTCCGGTTTG) | qRT-PCR (control) |
